# Auditory assessment and central auditory processing script for preschool children

**DOI:** 10.1590/2317-1782/20232021122en

**Published:** 2023-07-07

**Authors:** Isabelle Canevari de Albuquerque, Beatriz Servilha Brocchi

**Affiliations:** 1 Faculdade de Fonoaudiologia, Pontifícia Universidade Católica de Campinas - PUC-Campinas - Campinas (SP), Brasil.

**Keywords:** Audiology, Speech Therapy, Language Development, Child Development, Preschool

## Abstract

**Purpose:**

To develop an assessment script to observe hearing and central auditory processing in preschool children.

**Methods:**

To The script was prepared based on a search in the Scielo databases and in the library of a university in the state of São Paulo using the following keywords: “central auditory processing”, “hearing and language”, “auditory processing disorders”, “auditory processing in preschool children”, and “vocabulary assessment”, resulting in the selection of fourteen articles and two books. Then, questions related to auditory development and a script for assessing central auditory processing were prepared.

**Results:**

The script consists of eight parts, namely: Identification and Anamnesis, Information about Mother and Pregnancy, Complaints, Auditory Development, Language Development, Motor Development, Simplified Auditory Processing Evaluation and Behavioral Audiological Assessment.

**Conclusion:**

The script is essential, given the lack of screening instruments in the literature for central auditory processing in preschool children that thoroughly investigate the entire process that permeates the auditory and language development of children aged 43 to 47 months.

## INTRODUCTION

Hearing is widely understood as a prerequisite for the acquisition and development of oral language. In this sense, exposure to auditory experiences, especially in the first 3 years of life, which is considered a critical period of development, allows for the cortical organization that is necessary to ensure the normal development of hearing and language skills^( 1 )^. Hearing thresholds within the normal range and the proper functioning of central structures are essential for linguistic development^( 2 )^.

Another important factor is the peripheral auditory system, which is interconnected with the central auditory system. The peripheral auditory system comprises the outer, middle, inner ear and vestibulocochlear nerve, and is responsible for capturing, transmitting and transducing the sound wave and its processing in the cochlea and cochlear portion of the vestibulocochlear nerve^( 3 )^. In children with normal development, the cochlea is active from birth, unlike the central auditory system, which undergoes a maturation process through interference during childhood and adolescence^( 3 )^.

Auditory skills evolve from birth to two years of age, following a hierarchy^( 1 )^ through the following stages:

1. Detection: the ability to perceive the presence and absence of sounds, which occurs from the intrauterine stage;
2. Discrimination: the ability to differentiate two sounds the same or different, which is found even in newborns who can discriminate verbal sounds;
3. Localization: the ability to identify where the sound comes from, which is a stage that develops from four to 24 months;
4. Auditory recognition: the ability to associate signifier and meaning, pointing out figures or following orders;
5. Listening comprehension: the ability to understand speech, answer questions and retell stories.

According to these same authors, the ability to detect sounds has been present since intrauterine life, with the integrity of the peripheral auditory system, cochlea and acoustic nerve. Therefore, the ability to discriminate sounds can be observed in a newborn, when children differentiate their mothers' voices from the voices of other women^(1)^. In turn, sound localization occurs from four months of age and evolves as age increases. Sound localization begins with localization in the horizontal axis and progresses to the vertical position, from indirect to direct, then, later, there is localization in the longitudinal and transverse axis, involving the brainstem and cortex. Plasticity and maturation depend on stimulation, as specific neural pathways are activated and reinforced with the auditory experience, and neuroplasticity allows structural and functional changes to occur when there is stimulation^( 1 )^.

From the end of the first year of life, the auditory recognition ability emerges, evolving from simple to complex levels^( 1 )^. Children aged eight to ten months inhibit activities by recognizing when they hear the word “no”. Between nine and thirteen months, they recognize simple verbal commands, such as “to say goodbye” and from twelve months, children should be able to recognize their own names, which usually occurs from fifteen to eighteen months. From eighteen months to two years of age, auditory recognition skills evolve and children acquire story comprehension skills and the ability to answer questions about a story^( 1 )^.

In this context, the experiences and auditory situation in which the child is exposed are essential for the development and acquisition of language. During the process of maturation and plasticity it is essential to have a lot of stimulation to reinforce specific neural pathways. In this sense, some studies report that complications in language, speech and learning have been related to difficulty in processing acoustic stimuli^( 3 )^.

Thus, it is essential to know the functioning of the physiological mechanisms of the auditory system to have knowledge of auditory information processing, known as Central Auditory Processing (CAP). In addition, auditory disabilities must be identified early, as well as the relationship between these detected alterations and the learning of the language to which the child is exposed^( 2 )^.

The CAP refers to mechanisms and processes of the auditory system, which are responsible for sound localization, sound discrimination, and auditory recognition, temporal aspects of hearing involving resolution, masking, integration and temporal sequence, as well as auditory performance with concurrent acoustic signals and in unfavorable acoustic situations. These skills can be developed through verbal and non-verbal stimuli and, if altered, they may affect speech and language areas^( 4 )^.

The CAP assessment allows the verification of auditory behaviors and determines those that are within the process of evolution of a typical child and those that are deviant or with some level of disorder^( 2 )^. This assessment investigates auditory skills through behavioral observation of performance in different tasks, such as sound localization, temporal ordering, temporal resolution, dichotic task, monotic listening task and concurrent stimuli in the same ear^( 2 )^. Central Auditory Processing Disorder (CAPD) occurs when there is an impairment in auditory skills, even though the individual has normal auditory acuity and intelligence. Even if hearing is adequate, this disorder can be considered an inability to attend, discriminate, recognize, remember and understand information^( 5^. Changes can occur as a result of complications in developmental processes, and disorders impact speech perception, memory, language and language learning^( 2,6-8 )^.

Thus, early diagnosis in preschool children is essential to ensure that the intervention is carried out effectively. Given that failures in the development of preschool children have led to poor school performance, early detection made possible through screening and intervention is essential in the first five years of life^( 3 )^.

The literature shows the need for more research involving preschool children and investigating the diagnosis of auditory development and CAP so that early assessment can be performed. Early assessment allows the identification of possible changes in advance, thus resulting in less impact on the child's academic life.

### Purpose

This study aimed to develop a script for auditory assessment and CAP for preschool children.

## METHODS

The researchers prepared a script for auditory assessment and CAP (Appendix A) that is part of the project entitled “Association between auditory and vocabulary development of children aged 43 to 47 months”. This project was approved by the Research Ethics Committee PUC-Campinas under the Decision No. 3.426.010/2019. It should be noted that all participants signed the Free Prior Informed consent.

The researchers conducted a bibliographic search through the Scielo database and in a library using the following keywords in Brazilian Portuguese: “central auditory processing”, “hearing and language”, “auditory processing disorders”, “auditory processing in preschool children”, and “vocabulary assessment”. There were no limitations regarding the year and language for the selection of studies. The research found should follow some criteria to be included, such as: addressing the relationship between hearing and language, and addressing the main screening methods and CAP disorders.

In this context, 22 references were found that, after applying the criteria described above, were added to another 14 scientific articles and two books, as shown in Chart 1.

**Chart 1:** References used to prepare the research script

	Reference
Study	Mousinho R et al. Aquisição e desenvolvimento da linguagem: dificuldades que podem surgir neste percurso. Rev. Psicopedag. 2008; vol 25. Available from: http://pepsic.bvsalud.org/scielo.php?script=sci_arttext&pid=S0103-84862008000300012
Study	Capovilla, FC. Triagem de processamento auditivo central em crianças de 6 a 11 anos. Rev. Bras. Cresc. Des Hum. 2002; 12 (2). Available from: https://www.revistas.usp.br/jhgd/article/view/39692
Study	Souza IMP et al. Triagem do processamento auditivo central: contribuições do uso combinado de questionário e tarefas auditivas. Audiol Commun Res. 2018; vol 23. Available from: https://www.scielo.br/scielo.php?pid=S2317-64312018000100326&script=sci_abstract&tlng=pt
Study	Barry JG, Tomlin DM, David R, Dillon H. Use of Questionnaire-Based Measures in the Assessment of Listening Difficulties in School-Aged Children. Ear and Hearing. 2005; vol 36. Available from: https://www.ncbi.nlm.nih.gov/pmc/articles/PMC4617294/
Study	Schirmer CR, Fontoura DR, Nunes ML. Distúrbios da aquisição da linguagem e da aprendizagem. Soc Bras Ped. 2004; vol 80. Available from: https://www.scielo.br/scielo.php?script=sci_arttext&pid=S0021-75572004000300012#:~:text=Acredita%2Dse%20que%20as%20dificuldades,recep%C3%A7%C3%A3o%20verbal%20e%2Fou%20escrita.
Study	Luz DM, Costa-Ferreira MID. Identificação dos fatores de risco para o transtorno do processamento auditivo (central) em pré-escolares. Rev. CEFAC. 2011 fev; vol 13. Available from: https://www.scielo.br/scielo.php?script=sci_abstract&pid=S1516-18462011000400009&lng=pt&nrm=iso
Study	Simon LF, Rossi AG. Triagem do processamento auditivo em escolares de 8 a 10 anos. Psicol Esc Educ. 2006; vol 10. Available from: https://www.scielo.br/scielo.php?script=sci_arttext&pid=S1413-85572006000200012
Study	Caumo DTM, Ferreira MIDC. Relação entre desvios fonológicos e processamento auditivo. Rev soc bras fonoaudiol. 2009; vol 14. Available from: https://www.scielo.br/scielo.php?script=sci_arttext&pid=S1516-80342009000200015#:~:text=CONCLUS%C3%83O%3A%20A%20pesquisa%20sugere%20a,em%20crian%C3%A7as%20com%20desvio%20fonol%C3%B3gico.
Study	Machado CSS, Valle HLBS, Paula KM, Lima SS. Caracterização do processamento auditivo das crianças com distúrbio de leitura e escrita de 8 a 12 anos em tratamento no centro clínico de fonoaudiologia da pontifícia universidade católica de minas gerais. Rev Cefac. 2011; vol 13. Disponível em: https://www.scielo.br/scielo.php?pid=S1516-18462010005000119&script=sci_abstract&tlng=pt
Study	Oliveira AC, Cesar CPHAR, Matos GG, Pereira LD, Alves T, Guedes-Granzotti. Habilidades auditivas, de linguagem, motoras e sociais no desenvolvimento infantil: uma proposta de triagem. Rev Cefac. 2018; vol 20. Available from: https://www.scielo.br/pdf/rcefac/v20n2/pt_1982-0216-rcefac-20-02-00218.pdf
Study	Ferracini F, Capovilla AGS, Dias NM, Capovilla CF. Avaliação de vocabulário expressivo e receptivo na educação infantil. Rev Psicopedag. 2006; vol 23. Available from: http://pepsic.bvsalud.org/scielo.php?script=sci_arttext&pid=S0103-84862006000200006
Study	Kozlwski L, Wiemes GMR, Magni C, Silva ALG. A efetividade do treinamento auditivo na desordem do processamento auditivo central: estudo de caso. Rev. Bras. Otorrinolaringol. 2004; vol 70. Available from: https://www.scielo.br/scielo.php?pid=S0034-72992004000300023&script=sci_abstract&tlng=pt
Book	Boéchat EM, Menezes PL, Couto CM, Frizzo ACF, Scharlach RC, Anastasio ART. Tratado de Audiologia. 2 ed. Rio de Janeiro: Guanabara Koogan; 2015.
Study	Ferracini F, Capovilla AGS, Dias NM, Capovilla FC. Avaliação do vocabulario expressivo e receptivo na educação infantil. Rev Psicopedagogia. 2006. Available from: https://www.revistapsicopedagogia.com.br/detalhes/395/avaliacao-de-vocabulario-expressivo-e-receptivo-na-educacao-infantil#:~:text=Avalia%C3%A7%C3%A3o%20de%20vocabul%C3%A1rio%20expressivo%20e%20receptivo%20na%20educa%C3%A7%C3%A3o%20infantil,-Fernanda%20Ferracini1&text=Problemas%20com%20desenvolvimento%20da%20linguagem,risco%20de%20apresentar%20esses%20dist%C3%BArbios.
Study	Damazio M. Validação e normatização de instrumentos para avaliar vocabulários receptivo e expressivo em crianças de 18 meses a 6 anos de idade. Usp. 2015. Available from: https://teses.usp.br/teses/disponiveis/47/47132/tde-02032016-112936/pt-br.php
Book	Pereira LD, Schochat E. Testes auditivos comportamentais para avaliação do processamento auditivo central. Pró-fono; 2011.

At first, the script was structured by questions, from the first to the sixth topic, which were divided into objective and essay questions, totaling 68 questions. Then, in the second part of the script, which includes topics seven and eight, the script presents the protocols to be used to assess auditory development. The script must be completed by parents and guardians and, on average, takes 20 minutes to complete.

The assessment of CAP that is present in the script was based on the Simplified Auditory Processing Evaluation (SAPE)^( 9 )^, based on the book “Testes Auditivos Comportamentais para Avaliação” *[Behavioral Auditory Tests for Assessment]*, as a means of assessing sound localization, memory of verbal and non-verbal sounds. Since there are not enough resources in the literature for auditory assessment and CAP in preschool children, this instrument proved to be complete.

The script consisted of the following topics:

- Questions about participant identification and anamnesis, with essay and objective questions about information about the mother and the pregnancy.
- Behavioral auditory test for audiological assessment and Central Auditory Processing^( 9 )^.
- Behavioral audiological assessment using musical instruments.

For verification, the instrument was sent with a questionnaire for evaluation by three speech-language pathologists, two being language specialists and one audiologist. All three judges were scholars and had extensive experience in their respective fields.

The questionnaire submitted to the judges also included a brief introduction to the research, its objectives and methods. Then, five objective questions were elaborated, in which the judge should mark “yes” or “no”. In the last part, there was a free space for the judges to make observations regarding the script. All judges agreed with the removal of the memory of four syllables belonging to the ASPA^( 9 )^, used as a reference, as it was not expected for the age.

## RESULTS

The evaluation script consisted of eight parts, as described below, and can be found in Appendix A.

### Research script

#### Part 1: identification and anamnesis

The first part describes the child's main data, including name, age, sex, date of birth, place of birth, name of the school and session, and with whom they spend most of their time. In addition, this part also collects basic data information about family members, such as name and age, education level and amount of time they spend together for possible analysis of parent/child interaction.

#### Part 2: information about mother and pregnancy

The second part collects data regarding the mother and pregnancy, such as alcohol consumption; if the mother is a smoker and if she used medication. The questionnaire also includes questions about maternal diseases, such as HIV, syphilis, hypertension, diabetes mellitus, rubella, herpes, toxoplasmosis, cardiovascular disease and kidney disease. In addition, the questionnaire includes information on possible hospitalization and complications during pregnancy and at how many weeks the delivery took place.

These topics are directly associated with the health of the fetus during pregnancy. Any of these diseases can affect a child's healthy birth, including hearing and language.

#### Part 3: complaints

Part three of the questionnaire includes questions related to agitation, inattention; difficulty hearing and understanding in noisy environments; difficulty relating to people; and difficulties in performing global and fine motor activities, such as: “Does the child have complaints related to agitation?”, and “Does the child have difficulties in performing global motor activities?”.

#### Part 4: auditory development

In turn, part four, referring to auditory development, investigates cases of recurrent otitis in early childhood, whether there is otological history, otalgia, trauma, whether antibiotics were used and data on the family history of hearing loss, such as: “Does the child have a history of recurrent ear infections in early childhood?”, and “Does the child have frequent earaches?”.

#### Part 5: language development

The focus on the topic of language development is to analyze whether it is appropriate for the child's age. This topic investigates whether the preschool child is able to match three or more words, to identify body parts, or to understand instructions. In addition, the topic includes the following questions: “Is the child able to recognize adjectives such as ‘’big”, ‘’small”, and ‘’happy”?”; “Is the child able to use articles, such as “the” and “a”?”; “Is the child able to use plurals such as “candy” and “candies”?”; “Is the child able to use prepositions, such as “with”, “from”, “to”?”; and “Is the child able to use auxiliary verbs, such as “to have”, and “to be”?”.

In addition, the topic investigates the child's understanding of abstract concepts of quantity. The questionnaire also investigates whether the child is able to make deductions, formulate questions, requests actions, objects (such as giving a toy or getting water), permission (using “May I...”); uses negation, recounts past events, or anticipates the future. Other questions also assess whether the child is able to understand and is able to retell stories with narrative turns, keep a conversation topic waiting for their turn and whether they sing songs (“Is the child able to relate past events or anticipate the future?”, and “Is the child able to maintain a topic of conversation while waiting for his or her turn?”).

#### Part 6: motor development

Part six includes questions for parents about motor development.

#### Part 7: Simplified Auditory Processing Evaluation (SAPE)^( 9 )^

The initial part of the protocol collects data on the patient's name and age, the evaluator's name, and the evaluation date.

The first part of the protocol refers to the “sound localization test”, which investigates the child’s performance in right, left, top, front and back localization. According to the authors, the criterion of normality is to have four correct answers in five directions. The sound localization test table must be filled in with the number of correct answers and whether it is normal or altered. It should also be noted whether the assessment of the auditory ability of sound localization is normal or altered.

Part two begins the “Memory Test for Verbal or Non-Verbal Sounds in Sequence”, which is first subdivided into the Memory Test for Verbal Sounds (MTVS) in sequence with three sounds, in which the isolated phonoarticulatory production of the syllables PA TA CA must be carried out, and also fill in the table with “yes” or “no”, and the performance of the production of PA TA CA, TA PACA and CA TA PA. According to the authors, the criterion of normality in this case is to reach two or more correct answers in the MTVS in 3 attempts (≥2/3), and the result must be divided by three (as previously described, by consensus of the judges, the sequence of four sounds was removed from the script for this study).

The final part includes the completion of the questionnaire and must be filled in with the responses of the Memory Test for Verbal Sounds, including three verbal sounds in sequence. The same rule applies for the Memory Test for Non-Verbal Sounds, including three non-verbal sounds in sequence. The four non-verbal sounds test was withdrawn from the study, as it was considered to be inappropriate for the age group of the study. In this part of the study, the respondent must include the number of correct answers and whether it is normal or altered.

The respondent must also answer whether the assessment of the auditory temporal ordering ability is normal or altered. The following table should be filled in with the possible behaviors that were observed during the test, including: inadequate attention span, inadequate memory capacity, inadequate motor attitude, difficulty understanding requests and whether the child tires easily.

At the end of the test, the questionnaire details that the normality criterion for the sound localization test is to obtain more than four correct answers. As instructed by the authors, the evaluator must indicate the number of correct answers in a specific protocol, in addition to reporting whether the result is normal or changed (according to the score achieved). On the other hand, to reach the normality criterion in the sequence test for verbal sounds, the child must obtain at least two correct answers in the three named sequences, while the normality criterion is also to obtain two correct answers in three attempts for non-verbal sounds. Based on the findings of the three tests, the evaluator must conclude if there is a change in Central Auditory Processing. The four non-verbal sounds test was withdrawn from the study, as it was considered to be inappropriate for the age group of the study.

#### Part 8: behavioral audiological assessment

For the behavioral audiological assessment, the researchers developed a table based on the instruments that will be used, observing the child's auditory behavior according to the musical instrument. Thus, there is a table divided into instruments, intensity and responses. In this context, the instruments included are: drum, rattle, musical rattle, reco-reco and agogo with two bells. All these instruments should be tested at low, medium and strong intensity and the response must be recorded. The child at the age analyzed by the study is expected to respond to all musical instruments at the lowest intensity and directly in all directions.

Regarding the suggestions proposed by the judges, one of them proposed the use of the Language Development Assessment-LDA 2 protocol in the evaluation of preschool children. In addition, it was also suggested to include the educational level of the parents in the anamnesis. In the complaints, it was also suggested to change “handedness” to another topic, which is “identification and anamnesis”. For language development questions, include a question to see if the child can match four or more words.

It should be noted that little material regarding the CAP in preschool children was found in the research for the development of the script. On the other hand, there are many more studies involving school-age children, which shows the importance of material covering this age so that it is possible to detect early.

## DISCUSSION

As language shares underlying cognitive mechanisms with auditory skills, the integrity of the peripheral and central auditory system is critical to properly develop oral and written communication^( 10 )^. Some authors report that the first years of life are critical for development and that the auditory experiences during this period are directly linked to the development of hearing skills, such as detection, discrimination, location, recognition and listening comprehension^( 11 )^. In this way, the neuromuscular and sensory system maturation in an integral way is directly linked to speech and language skills. The auditory ability of speech perception arises when an articulatory pattern of language is acquired, as the sensory and motor aspects are being covered.

Central auditory processing, which develops until, on average, ten to twelve years of age, is the mechanism responsible for auditory skills^( 11 )^. There are factors, such as the integrity of the peripheral auditory system and the maturation of the central nervous system, especially with regard to the primary and secondary auditory areas that can directly influence auditory development^( 12 )^. These factors must be extensively investigated to understand possible changes. Thus, the script aimed to thoroughly investigate the main risk factors for changes in the CAP.

Peripheral alterations and history of secretory otitis media during early childhood can compromise the maturation of the auditory pathways. This impairment has an impact on central auditory skills and the learning process, hence the need to investigate the patient's auditory history^( 10 )^.

The questions about the child's auditory development, history, if there was any trauma, family history and if he/she used antibiotics that could be ototoxic are addressed in part four of the script, which is called “auditory development”. According to the literature, the etiology of CAP disorders includes otitis, high and continuous fevers, specific disorders of the development of auditory function, damage to the conduction pathways and sensory deprivation during early childhood^( 13 )^.

All these factors mentioned above will effectively impact the auditory development, as well as the patient's oral and written language. Difficulties can be observed by family members and caregivers through behaviors, such as difficulties in performing complex or longer tasks, distraction, sensitivity to loud sounds, difficulty following verbal orders, repetition of verbal stimuli, difficulty understanding jokes and language figurative^( 5 )^. The complaints were addressed in part three of the script, as the difficulty in understanding speech in the presence of background noise, greater distractibility, reduced attention, communication difficulties and low academic performance are signs of risk CAP for disorders^( 13 )^.

Auditory processing disorders consist of an inability to attend to, discriminate, recognize, remember, or understand auditory information^( 11 )^.

If any impairment is found in the areas described above, there is a need for an in-depth assessment. It is important to assess skills that are part of normal language development, as many oral language disorders can be detected and followed up early.

Part five of the script, characterized as “language development”, addresses the main development milestones. Impairments in the interpretation of sensory information may be indicative of hearing impairment, which, in the future, may lead to an auditory processing disorder, and in the acquisition of speech and language^( 12 )^.

According to the researched literature, there is no instrument that investigates the relationship between language and hearing, especially CAP in preschool children, only in school-age children. The researchers searched for articles and materials on the subject in the Lilacs, SciELO, PubMed databases and in the collection of books and theses in the institution's library, but only protocols and scripts that included children over six years of age were found. Thus, it is essential to develop material that aims at the early screening of risks and possible hearing and language alterations.

Therefore, this script is an instrument to be used in clinical practice for assessments, screening and monitoring of preschool children to indicate possible risks for auditory information processing disorder. This study aimed at early detection of possible disorders for monitoring and stimulation of auditory skills so that the damage can be remedied or reduced for the child.

The script is part of an ongoing study that will evaluate children under the age of six. The research has not yet taken place due to the COVID-19 pandemic.

## CONCLUSION

The researchers prepared a research script to carry out an assessment and find possible risks for CAP disorder in preschool children. The script is essential, given the lack of screening instruments in the literature for CAP in preschool children that thoroughly investigate the entire process that permeates the auditory and language development of children aged 43 to 47 months.

The period selected for the investigation is critical for development, and some factors mentioned, such as otitis and the use of ototoxic drugs, among others, can cause changes in auditory development that impact the development of oral and written language and school learning.

## Appendix A Assessment script

1. Identification and Anamnesis

Name:

Age:

Female () Male ()

Date of Birth:

Place of Birth:

Phone No.:

School: Session:

Handedness:

Who does the child spend most of the time with?

Mother: Age:

Profession:

Educational level:

Time spent with the child per day:

Father: Age:

Profession:

Educational level:

Time spent with the child per day:

2. Information about Mother and Pregnancy

Smoking () Yes () No

Alcohol consumption () Yes () No

Use of Medication:

HIV () Yes () No

Syphilis () Yes () No

Hypertension () Yes () No

Diabetes mellitus () Yes () No

Rubella () Yes () No

Herpes () Yes () No

Toxoplasmosis () Yes () No

Cardiovascular diseases () Yes () No

Kidney diseases () Yes () No

Hospitalization during pregnancy:

Complications:

How many weeks of pregnancy was the baby born?

Notes:

3. Complaints

Does the child have complaints related to agitation?

Does the child have complaints related to inattention?

Does the child have difficulty hearing and understanding in noisy environments?

Does the child have difficulty relating to people?

Does the child have difficulties in performing global motor activities?

Does the child have difficulties in performing fine motor activities?

4. Auditory Development:

Does the child have a history of recurrent ear infections in early childhood?

Does the child have a history of otological problems?

Does the child have frequent earaches **(otalgia)**?

Has the child ever had any trauma to the ear?

Does the child use antibiotics? **If yes, when and for how long?**

Family history?

5. Language Development

Is the child able to match three or more words?

Is the child able to identify body parts?

Is the child able to understand instructions received?

Is the child able to recognize pronouns that differentiate between the sexes?

Is the child able to recognize adjectives such as ‘’big”, ‘’small”, and ‘’happy”?

Is the child able to use articles, such as “the” and “a”?

Is the child able to use plurals such as “candy” and “candies”?

Is the child able to use prepositions, such as “with”, “from”, “to”?

Is the child able to use auxiliary verbs, such as “to have”, and “to be”?

Is the child able to understand abstract concepts?

Is the child able to understand the concept of quantity?

Is the child able to make deductions?

Is the child able to ask questions?

Is the child able to request actions and objects, such as giving a toy or getting water?

Is the child able to ask permission by saying “may I...”?

Is the child able to use denial?

Is the child able to relate past events or anticipate the future?

Is the child able to understand and retell stories with narrative turns?

Is the child able to maintain a topic of conversation while waiting for his or her turn?

Is the child able to sing songs?

6. Motor Development

Is the child's motor development appropriate for their age?

7. Simplified Auditory Processing Evaluation (SAPE)

Name:

Age:

Name of Evaluator:

Evaluation Date:

1. Sound Localization Test

Performance

() Right () Left () Up () Front () Back

Normality Criterion

≥4 correct answers including right and left

Result: _____

**Table:**

	Correct Answers	Normal	Changed
Sound Localization Test			

Assessment of the auditory ability of sound localization:

() Normal () Changed

2. Memory Test for Verbal or Non-Verbal Sounds in Sequence

2.1 MTVS in Sequence (using three sounds):

Isolated phonoarticulatory production of the syllable () PA () TA () CA

Performance:

**Table:**

	Yes	No
PA TA CA		
TA PA CA		
CA TA PA		

Normality Criterion

≥2 correct answers for the MTVS in 3 attempts (≥2/3)

Result: ____ (using three sounds)

Conclusion

**Table:**

	Correct Answers	Normal	Changed

Assessment of auditory temporal ordering ability:

() Normal () Changed

Other behaviors observed during this test:

() Inadequate attention span

() Inadequate memory capacity

() Inadequate motor behavior

() Difficulty understanding requests

() The child gets tired easily

Notes: ___________________________________________________________________________________________________________________________

For the sound localization test, the normality criterion is to obtain more than four correct answers. The evaluator must indicate the number of correct answers in a specific protocol, in addition to reporting whether the result is normal or changed (according to the score achieved). On the other hand, to reach the normality criterion in the sequence test for verbal sounds, the child must obtain at least two correct answers in the three named sequences, while the normality criterion is also to obtain two correct answers in three attempts for non-verbal sounds. Based on the findings of the three tests, the evaluator must conclude if there is a change in Central Auditory Processing.

Adequate: () Yes () No

8. Behavioral Audiological Assessment:

Observed through the child's auditory behavior before the instrumental stimulus.

**Table:**

Instrument	Intensity	Response
Drum	Low ()	
	Medium ()	
	High ()	
Rattle	Low ()	
	Medium ()	
	High ()	
Musical rattle	Low ()	
	Medium ()	
	High ()	
Reco-reco	Low ()	
	Medium ()	
	High ()	
Agogo (two bells)	Low ()	
	Medium ()	
	High ()	

Adequate: () Yes () No

Cochleopalpebral reflex for intense sounds (drum and agogo)

Adequate: () Yes () No

## References

[1] Azevedo MF, Bevilacqua MC, Martinez MAN, Balen SA, Pupo AC, Reis ACMB, Frota S (2011). Tratado de audiologia.

[2] Pereira LD, Interamerican Association of Pediatric Otorhinolaryngology (2015). V manual de otorrinolaringologia pediátrica da IAPO.

[3] Carvalho ND, Novelli CVL, Colella-Santos MF (2015). Fatores na infância e adolescência que podem influenciar o processamento auditivo: revisão sistemática. Rev CEFAC.

[4] Kozlowski L, Wiemes GMR, Magni C, Silva ALG (2004). A efetividade do treinamento auditivo na desordem do processamento auditivo central: estudo de caso. Rev Bras Otorrinolaringol.

[5] Simon LF, Rossi AG (2006). Triagem do processamento auditivo em escolares de 8 a 10 anos. Psicol Esc Educ.

[6] Moore DR, Ferguson MA, Edmondson-Jones AM, Ratib S, Riley A (2010). Nature of auditory processing disorder in children. Pediatrics.

[7] Ahmmed AU, Ahmmed AA, Bath JR, Ferguson MA, Plack CJ, Moore DR (2014). Assessment of children with suspected auditory processing disorder: a factor analysis study. Ear Hear.

[8] Carvalho NG, Ubiali T, Amaral MIR, Colella-Santos MF (2019). Procedures for central auditory processing screening in schoolchildren. Braz J Otorhinolaryngol.

[9] Pereira LD, Pereira LD, Schochat E (1997). Processamento auditivo central: manual de avaliação.

[10] Souza IMP, Carvalho NG, Plotegher SDCB, Colella-Santos MF, Amaral MIR (2018). Triagem do processamento auditivo central: contribuições do uso combinado de questionário e tarefas auditivas. Audiol Commun Res.

[11] Azevedo MF, Angrissani RG, Boéchat E, Menezes PL, Couto CM, Frizzo ACF, Scharlach RC, Anastásio ART (2015). Tratado de audiologia.

[12] Caumo DTM, Ferreira MIDC (2009). Relação entre desvios fonológicos e processamento auditivo. Rev Soc Bras Fonoaudiol.

[13] Capovilla FC (2002). Triagem de processamento auditivo central em crianças de 6 a 11 anos. J Hum Growth Dev.

