# Supplementary material for: Auditory assessment and central auditory processing script for preschool children
Source: Codas. 2023 Jul 7;35(3):e20210122. doi: 10.1590/2317-1782/20232021122en (PMC10348662; doi:10.1590/2317-1782/20232021122en)
Supplement: Translation

# Translation: Roteiro de avaliação auditiva e do processamento auditivo central para pré-escolares

**DOI:** 10.1590/2317-1782/20232021122pt

**Authors:** Isabelle Canevari de Albuquerque, Beatriz Servilha Brocchi

**Affiliations:** 1 Faculdade de Fonoaudiologia, Pontifícia Universidade Católica de Campinas - PUC-Campinas - Campinas (SP), Brasil.

**Keywords:** Audiologia, Fonoaudiologia, Desenvolvimento da Linguagem, Desenvolvimento Infantil, Pré-escolar

## Abstract

**Objetivo:**

Construir um roteiro de avaliação para observação da audição e do processamento auditivo central em pré-escolares.

**Método:**

Para elaboração do roteiro, primeiramente realizou-se uma busca nas bases de dados Scielo e biblioteca de uma universidade no estado de São Paulo, com as palavras chaves: “processamento auditivo central”, “audição e linguagem”, “distúrbios do processamento auditivo”, “processamento auditivo em pré-escolares”, “avaliação do vocabulário”, selecionando então quatorze artigos e dois livros. Foram estruturadas perguntas relacionadas ao desenvolvimento auditivo e um roteiro de avaliação do processamento auditivo central.

**Resultados:**

O roteiro constitui-se de oito partes, sendo identificação e anamnese, dados maternos e de gestação, queixas, desenvolvimento auditivo, desenvolvimento de linguagem, desenvolvimento motor, Teste auditivo comportamental para avaliação do processamento auditivo central e avaliação audiológica comportamental.

**Conclusão:**

O roteiro é de extrema importância visto que não há na literatura instrumentos de triagem de processamento auditivo em pré-escolares que investigue, de forma minuciosa, todo o processo que permeia o desenvolvimento auditivo e de linguagem de crianças de 43 a 47 meses.

## INTRODUÇÃO

A audição é considerada um pré-requisito para a aquisição e desenvolvimento da linguagem oral. A exposição às experiências auditivas, principalmente nos três primeiros anos de vida, considerado período crítico do desenvolvimento, permite a organização cortical necessária para garantir o desenvolvimento normal da audição e da linguagem^(1)^. Os limiares auditivos dentro dos padrões de normalidade e o funcionamento adequado das estruturas centrais são de fundamental importância para o desenvolvimento linguístico^(2)^.

O sistema auditivo periférico está interligado com o sistema auditivo central. O sistema auditivo periférico abrange a orelha externa, média, interna e nervo vestibulococlear, tendo como função captar, transmitir e realizar transdução da onda sonora e seu processamento na cóclea e porção coclear do nervo vestibulococlear^(3)^. Em crianças com desenvolvimento dentro do padrão de normalidade, a cóclea está em ação desde o nascimento, diferente do sistema auditivo central que passa por um processo de amadurecimento através das interferências no período da infância e adolescência^(3)^.

As habilidades auditivas evoluem desde o nascimento até os dois anos de idade, seguindo uma hierarquia^(1)^. As etapas podem ser consideradas como:

1. Detecção: perceber presença e ausência de sons, ocorre desde intraútero;
2. Discriminação: diferenciação de dois sons iguais ou diferentes, recém-nascidos discriminam sons verbais;
3. Localização: identificar de onde vem o som, essa etapa se desenvolve dos quatro aos 24 meses;
4. Reconhecimento auditivo: associação do significante e significado, apontando figuras ou cumprindo ordens;
5. Compreensão auditiva: entender falas, responder perguntas e recontar histórias.

Segundo os mesmos autores, desde a vida intrauterina, com integridade do sistema auditivo periférico, cóclea e nervo acústico, a habilidade de detectar sons já está presente. A habilidade de discriminação de sons pode ser observada em um recém-nascido, em que a criança diferencia a voz da sua mãe de outras mulheres^(1)^. A localização sonora, por sua vez, ocorre a partir de quatro meses e evolui conforme a idade aumenta. Inicia-se por localização no eixo horizontal e progride para a posição vertical, da maneira indireta para direta. Posteriormente, há a localização no eixo longitudinal e transversal, envolvendo tronco encefálico e córtex. A plasticidade e maturação são dependentes de estimulação, visto que adicionado experiência auditiva as vias neurais específicas são ativadas e reforçadas, logo, a neuroplasticidade permite que ocorram mudanças estruturais e funcionais diante à estimulação^(1)^.

A habilidade de reconhecimento auditivo surge no final do primeiro ano de vida, evoluindo dos níveis simples para complexos^(1)^. Crianças de oito a dez meses inibem atividades ao reconhecer quando a palavra ‘’não” é falada; entre nove e 13 meses, reconhecem comandos verbais simples como ‘’dá tchau” e, a partir de 12 meses, a criança deve reconhecer o próprio nome ocorrendo geralmente dos 15 aos 18 meses. Dos 18 meses a dois anos de idade, a habilidade de reconhecimento auditivo evolui para compreensão de histórias e habilidades de responder perguntas sobre uma história^(1)^.

Uma condição necessária para o desenvolvimento e aquisição da linguagem são as experiências e situações auditivas em que a criança fica exposta. É imprescindível que haja muita estimulação durante o processo de maturação e plasticidade para reforçar as vias neurais específicas. Estudos demonstram que complicações na linguagem, fala e aprendizado tem sido relacionadas com dificuldade no processamento dos estímulos acústicos^(3)^.

Desta forma, conhecer o funcionamento dos mecanismos fisiológicos do sistema auditivo é fundamental para o conhecimento do processamento da informação auditiva, conhecido como Processamento Auditivo Central (PAC). Além disso, é de grande importância reconhecer inabilidades auditivas precocemente, bem como a relação entre essas alterações detectadas e a aprendizagem da língua em que a criança está exposta^(2)^.

O PAC refere-se a mecanismos e processos do sistema auditivo, que são responsáveis pela localização sonora, discriminação sonora, reconhecimento auditivo, aspectos temporais da audição envolvendo resolução, mascaramento, integração e sequência temporal, também pelo desempenho auditivo com sinais acústicos em competição e em situações acústicas desfavoráveis. Essas habilidades podem ser por estímulos verbais e não-verbais e, se alteradas, afetam áreas de fala e linguagem^(4)^.

A avaliação do PAC permite a verificação dos comportamentos auditivos e determina aqueles que estão dentro do processo de evolução de uma criança típica e aqueles que estão desviados ou com algum nível de distúrbio^(2)^. Trata-se de uma avaliação das habilidades auditivas por meio de observação comportamental do desempenho frente a diferentes tarefas, tais como localização sonora, ordenação temporal, resolução temporal, tarefa dicótica, tarefa de escuta monótica e estímulos competitivos na mesma orelha^(2)^. Quando há um impedimento nas habilidades auditivas, mesmo que o indivíduo possua acuidade auditiva e inteligência normal, ocorre o Transtorno do Processamento Auditivo Central (TPAC). Esse transtorno pode ser considerado uma inabilidade de atender, discriminar, reconhecer, recordar e compreender informações mesmo que a audição esteja adequada^(5)^. Alterações podem ocorrer por intercorrências nos processos de desenvolvimento. Os transtornos trazem repercussões na percepção de fala, memória, linguagem e aprendizado da linguagem^(2,6-8)^.

O diagnóstico precoce é de extrema importância em pré-escolares para que a intervenção seja feita de maneira efetiva. Falhas no desenvolvimento de pré-escolares acarretaram um desempenho escolar inadequado, logo detecção precoces por meio de triagens e a realização de intervenções é essencial nos primeiros cinco anos^(3)^.

Nota-se na literatura a necessidade de mais pesquisas em pré-escolares sobre o diagnóstico do desenvolvimento auditivo e PAC para que ocorra avaliação precoce. Através dela possíveis alterações podem ser identificadas previamente causando menor impacto na vida acadêmica da criança.

### Objetivos

O objetivo geral é elaborar um roteiro de avaliação auditiva e PAC para crianças pré-escolares.

## MÉTODO

Foi realizada a elaboração de um roteiro de avaliação auditiva e do PAC (Anexo A) que faz parte do projeto intitulado “Relação entre o desenvolvimento auditivo e de vocabulário de crianças de 43 a 47 meses de idade”. Este projeto possui consentimento do Comitê de Ética da PUC-Campinas para realizá-lo, CEP 3.426.010/2019. Todos os indivíduos envolvidos assinaram o Termo de Consentimento Livre e Esclarecido.

Realizou-se uma pesquisa bibliográfica através da base de dados Scielo e biblioteca pertencente a uma instituição utilizando as palavras-chaves: ‘’processamento auditivo central”, ‘’audição e linguagem”, ‘’distúrbios do processamento auditivo”, ‘’processamento auditivo em pré-escolares”, ‘’avaliação do vocabulário”. Não houve limitações quanto ao ano e idioma para a seleção. As pesquisas encontradas deveriam seguir critérios para serem incluídas, como: abordar a relação entre a audição e a linguagem, abordar os principais métodos de triagem do e os transtornos do PAC.

Foram encontradas 22 referências, mas aplicando os critérios acima descritos, foram incluídos 14 artigos científicos e dois livros apresentados no Quadro 1.

**Quadro 1:** Referências utilizadas para a elaboração do roteiro de pesquisa

	Referências
Artigo	Mousinho R et al. Aquisição e desenvolvimento da linguagem: dificuldades que podem surgir neste percurso. Rev. Psicopedag. 2008; vol 25. Disponível em: http://pepsic.bvsalud.org/scielo.php?script=sci_arttext&pid=S0103-84862008000300012
Artigo	Capovilla, FC. Triagem de processamento auditivo central em crianças de 6 a 11 anos. Rev. Bras. Cresc. Des Hum. 2002; 12 (2). Disponível em: https://www.revistas.usp.br/jhgd/article/view/39692
Artigo	Souza IMP et al. Triagem do processamento auditivo central: contribuições do uso combinado de questionário e tarefas auditivas. Audiol Commun Res. 2018; vol 23. Disponível em: https://www.scielo.br/scielo.php?pid=S2317-64312018000100326&script=sci_abstract&tlng=pt
Artigo	Barry JG, Tomlin DM, David R, Dillon H. Use of Questionnaire-Based Measures in the Assessment of Listening Difficulties in School-Aged Children. Ear and Hearing. 2005; vol 36. Disponível em: https://www.ncbi.nlm.nih.gov/pmc/articles/PMC4617294/
Artigo	Schirmer CR, Fontoura DR, Nunes ML. Distúrbios da aquisição da linguagem e da aprendizagem. Soc Bras Ped. 2004; vol 80. Disponível em: https://www.scielo.br/scielo.php?script=sci_arttext&pid=S0021-75572004000300012#:~:text=Acredita%2Dse%20que%20as%20dificuldades,recep%C3%A7%C3%A3o%20verbal%20e%2Fou%20escrita.
Artigo	Luz DM, Costa-Ferreira MID. Identificação dos fatores de risco para o transtorno do processamento auditivo (central) em pré-escolares. Rev. CEFAC. 2011 fev; vol 13. Disponível em: https://www.scielo.br/scielo.php?script=sci_abstract&pid=S1516-18462011000400009&lng=pt&nrm=iso
Artigo	Simon LF, Rossi AG. Triagem do processamento auditivo em escolares de 8 a 10 anos. Psicol Esc Educ. 2006; vol 10. Disponível em: https://www.scielo.br/scielo.php?script=sci_arttext&pid=S1413-85572006000200012
Artigo	Caumo DTM, Ferreira MIDC. Relação entre desvios fonológicos e processamento auditivo. Rev soc bras fonoaudiol. 2009; vol 14. Disponível em: https://www.scielo.br/scielo.php?script=sci_arttext&pid=S1516-80342009000200015#:~:text=CONCLUS%C3%83O%3A%20A%20pesquisa%20sugere%20a,em%20crian%C3%A7as%20com%20desvio%20fonol%C3%B3gico.
Artigo	Machado CSS, Valle HLBS, Paula KM, Lima SS. Caracterização do processamento auditivo das crianças com distúrbio de leitura e escrita de 8 a 12 anos em tratamento no centro clínico de fonoaudiologia da pontifícia universidade católica de minas gerais. Rev Cefac. 2011; vol 13. Disponível em: https://www.scielo.br/scielo.php?pid=S1516-18462010005000119&script=sci_abstract&tlng=pt
Artigo	Oliveira AC, Cesar CPHAR, Matos GG, Pereira LD, Alves T, Guedes-Granzotti. Habilidades auditivas, de linguagem, motoras e sociais no desenvolvimento infantil: uma proposta de triagem. Rev Cefac. 2018; vol 20. Disponível em: https://www.scielo.br/pdf/rcefac/v20n2/pt_1982-0216-rcefac-20-02-00218.pdf
Artigo	Ferracini F, Capovilla AGS, Dias NM, Capovilla CF. Avaliação de vocabulário expressivo e receptivo na educação infantil. Rev Psicopedag. 2006; vol 23. Disponível em: http://pepsic.bvsalud.org/scielo.php?script=sci_arttext&pid=S0103-84862006000200006
Artigo	Kozlowski L, Wiemes GMR, Magni C, Silva ALG. A efetividade do treinamento auditivo na desordem do processamento auditivo central: estudo de caso. Rev. Bras. Otorrinolaringol. 2004; vol 70. Disponível em: https://www.scielo.br/scielo.php?pid=S0034-72992004000300023&script=sci_abstract&tlng=pt
Livro	Boéchat EM, Menezes PL, Couto CM, Frizzo ACF, Scharlach RC, Anastasio ART. Tratado de Audiologia. 2 ed. Rio de Janeiro: Guanabara Koogan; 2015.
Artigo	Ferracini F, Capovilla AGS, Dias NM, Capovilla FC. Avaliação do vocabulario expressivo e receptivo na educação infantil. Rev Psicopedagogia. 2006. Disponível em: https://www.revistapsicopedagogia.com.br/detalhes/395/avaliacao-de-vocabulario-expressivo-e-receptivo-na-educacao-infantil#:~:text=Avalia%C3%A7%C3%A3o%20de%20vocabul%C3%A1rio%20expressivo%20e%20receptivo%20na%20educa%C3%A7%C3%A3o%20infantil,-Fernanda%20Ferracini1&text=Problemas%20com%20desenvolvimento%20da%20linguagem,risco%20de%20apresentar%20esses%20dist%C3%BArbios.
Artigo	Damazio M. Validação e normatização de instrumentos para avaliar vocabulários receptivo e expressivo em crianças de 18 meses a 6 anos de idade. Usp. 2015. Disponível em: https://teses.usp.br/teses/disponiveis/47/47132/tde-02032016-112936/pt-br.php
Livro	Pereira LD, Schochat E. Testes auditivos comportamentais para avaliação do processamento auditivo central. Pró-fono; 2011.

O roteiro foi estruturado, primeiramente, por perguntas, do primeiro ao sexto tópico, divididas em objetivas e dissertativas, totalizando 68 questões. Em seguida, na segunda parte do roteiro, do tópico sete e oito, estão configurados os protocolos a serem utilizados para avaliação do desenvolvimento auditivo. O roteiro é destinado para pais e responsáveis responderem, tendo tempo médio de duração de 20 minutos.

A avaliação do PAC, presente no roteiro, foi baseada na Avaliação Simplificada do Processamento Auditivo (ASPA)^(9)^. Retirado do livro ‘’Testes Auditivos Comportamentais para Avaliação”, como meio de avaliação de localização sonora, memória de sons verbais e não verbais. Esse instrumento mostrou-se completo visto que não há na literatura recursos suficientes para avaliação auditiva e do PAC de pré-escolares.

O roteiro foi constituído dos seguintes tópicos:

- Perguntas sobre identificação do participante e anamnese, com questões dissertativas e objetivas sobre os dados maternos e de gestação.
- Teste auditivo comportamental para avaliação audiológica e do PAC^(9)^.
- Avaliação audiológica comportamental por meio de instrumentos musicais.

Para verificação do instrumento, o mesmo foi encaminhado com um questionário para avaliação de coerência de três juízes, fonoaudiólogos, dois especialistas em linguagem e um audiologista. Todos são acadêmicos e possuem vasta experiência em suas respectivas áreas.

O questionário enviado aos juízes possui uma breve introdução da pesquisa, seus objetivos e métodos. Em seguida, foram elaboradas cinco perguntas objetivas, em que o juiz assinalou ‘’sim” ou ‘’não”. Na última parte, havia um espaço para observações pertinentes ao roteiro. Todos concordaram com a retirada da memória de quatro sílabas pertencente ao ASPA^(9)^, usado como referência, pois não era esperado para a idade.

## RESULTADOS

O roteiro de avaliação está constituído por oito partes descritas a seguir e encontra-se no Anexo A.

### Roteiro de pesquisa

#### Parte 1: identificação e anamnese

Na primeira parte há os principais dados da criança, incluindo nome, idade, sexo feminino ou masculino, data de nascimento, naturalidade, escola em que está matriculada e período e com quem passa a maior parte do seu tempo. Há também dados básicos sobre os familiares, sendo nome e idade, grau de escolaridade e quantidade de tempo que passam juntos para possível análise de interação pais/criança.

#### Parte 2: dados maternos e de gestação

Na segunda parte encontram-se os dados referentes a mãe e a gestação, como ingestão de bebida alcoólica; se a mãe é tabagista e se fez uso de medicamentos. Há também perguntas sobre doenças maternas como HIV, sífilis, hipertensão, diabetes, rubéola, herpes, toxoplasmose, doenças cardiovasculares e doenças renais. Além disso, se ocorreu internação e intercorrências durante a gestação e com quantas semanas o parto foi realizado.

Estes tópicos possuem ligação direta com a saúde do feto durante a gestação. Qualquer uma dessas doenças pode afetar o nascimento saudável da criança, incluindo audição e linguagem.

#### Parte 3: queixas

Na parte três, há questionamentos relacionados à agitação, desatenção; dificuldade para ouvir e entender em ambientes ruidosos; dificuldade para se relacionar com as pessoas; dificuldades para realizar atividades motoras globais e finas, como, por exemplo: ‘’apresenta queixas relacionadas a agitação?”, ‘’apresenta dificuldade para realizar atividades motoras globais?”.

#### Parte 4: desenvolvimento auditivo

Na parte quatro, referente ao desenvolvimento auditivo, investiga-se casos de otites recorrentes na primeira infância, se há antecedentes otológicos, otalgia, trauma, se fez uso de antibióticos e qual o histórico familiar em relação a perdas auditivas, como: ‘’histórico de otites recorrentes na primeira infância?”, ‘’sente dores frequentemente?”.

#### Parte 5: desenvolvimento da linguagem

No tópico de desenvolvimento de linguagem, o foco é analisar se o mesmo está adequado para a idade da criança. Questiona-se se o pré-escolar faz combinação de três ou mais palavras, identifica as partes do corpo, compreende instruções que lhe são direcionadas. Reconhece pronomes que diferenciam os sexos; reconhece adjetivos, como ‘’grande”, ‘’pequeno”, ‘’feliz”; faz uso de artigos, como ‘’o”, ‘’a”, ‘’um”, ‘’uma”; faz uso de plurais, como ‘’bala-balas’; faz uso de preposições, como ‘’com”, ‘’de”, ‘’para” e faz uso de verbos auxiliares, como ‘’ ter”, ‘’ser”, ‘’estar”.

Além disso, observa-se a compreensão de conceitos abstratos, de quantidade. Nota-se também se a criança faz deduções, formula perguntas, solicita ações, objetos (como dar um brinquedo ou pegar água), permissão (com ‘’posso”); usa a negação, relata acontecimentos do passado ou antecipa do futuro. Compreende e consegue recontar histórias com turnos narrativos, tópico de conversação aguardando seu turno e se canta músicas (‘’relata acontecimentos do passado ou antecipa do futuro?”, ‘’mantém tópico de conversação aguardando seu turno?”).

#### Parte 6: desenvolvimento motor

Na parte seis, pergunta-se aos pais sobre desenvolvimento motor.

#### Parte 7: Avaliação Simplificada do Processamento Auditivo (ASPA)^(9)^

O protocolo tem início com o nome do paciente e a idade, o nome do avaliador e a data de avaliação.

A primeira parte do protocolo refere-se ao ‘’teste de localização sonora”, o qual investiga o desempenho da criança na localização à direita, à esquerda, em cima, à frente e atrás. O critério de normalidade, de acordo com os autores, é acertar quatro de cinco direções. A tabela do teste de localização sonora deve ser preenchida com a quantidade de acertos e se está normal ou alterado. Deve-se também assinalar se a avaliação da habilidade auditiva de localização sonora encontra-se normal ou alterada.

Na parte dois se inicia o ‘’Teste de memória para sons verbais ou não verbais em sequência”, subdividido primeiro em Teste de Memória para Sons Verbais (TMSV) em sequência com três sons, em que deve ser realizada a produção fonoarticulatória isolada das sílabas PA TA CA, e também preencher na tabela com ‘’sim” ou ‘’não”, o desempenho da produção do PA TA CA, TA PA CA e CA TA PA. O critério de normalidade, segundos os autores, é igual ou maior a dois acertos para TMSV em 3 tentativas (≥2/3), o resultado deve ser dividido por três (como descrito anteriormente, por consenso dos juízes da pesquisa, a sequência de quatro sons foi retirada do roteiro para o presente estudo).

Na parte final há a conclusão, deve ser preenchido com as respostas do teste de memória sequencial de sons verbais, incluindo três sons verbais. Aplica-se a mesma regra para o teste de memória sequencial de sons não verbais, incluindo três sons não verbais. O teste de quatro sons não verbais foi retirado pois não estava adequado para a faixa etária do estudo. Neste momento é colocado os acertos e se está normal ou alterado.

Deve preencher se a avaliação da habilidade auditiva de ordenação temporal está normal ou alterada. A tabela seguinte é preenchida com os possíveis comportamentos que foram observados durante a realização do teste, incluindo: capacidade de atenção inadequada, capacidade de memória inadequada, atitude motora inadequada, dificuldade de compreender as solicitações e cansa-se facilmente.

Finalizando o teste, é explicado que, para o teste de localização sonora, é colocado como critério de normalidade, acima de quatro acertos. Como orientado pelas autoras, o avaliador deverá marcar, em protocolo específico, o número de acertos e se está normal ou alterado (de acordo com a pontuação alcançada). Para o teste de sequência para sons verbais deverá haver ao menos dois acertos nas três sequências nomeadas como critério de normalidade, assim como para sons não verbais, em que deverá haver também dois acertos em três tentativas. Com os achados dos três testes, o avaliador concluirá se há alteração de PAC. O teste de quatro sons foi retirado pois não se apresentava adequado para a idade do presente estudo.

#### Parte 8: avaliação audiológica comportamental

Em avaliação audiológica comportamental, foi construída uma tabela a partir dos instrumentos que serão utilizados, observando o comportamento auditivo da criança diante do instrumento musical. Há uma tabela dividida em instrumentos, intensidade e respostas. Os instrumentos serão: tambor, chocalho, guizo, reco-reco e agogô de duas campânulas, todos devem ser testados em fraco, médio e forte e a resposta deve ser anotada. É esperado que a criança na idade analisada pelo estudo responda a todos os instrumentos musicais na intensidade mais fraca e diretamente para todas as direções.

Em relação às sugestões propostas pelos juízes, um deles propôs que o protocolo Avaliação do Desenvolvimento da Linguagem- ADL 2 pudesse ser utilizado na avaliação dos pré-escolares. Foi sugerido incluir, na anamnese, grau de instrução/escolaridade dos pais. Nas queixas, foi sugerido também retirar ‘’preferência manual” para ser inserido em outro tópico, sendo este ‘’identificação e anamnese”. Nas questões de desenvolvimento de linguagem, incluir uma pergunta para saber se a criança combina quatro palavras ou mais.

Na pesquisa para construção do roteiro foi encontrado pouco material referente ao PAC em pré-escolares. Já em escolares o foco de estudos é maior, mostrando a importância de um material que abrange essa idade para que seja possível detectar de maneira precoce.

## DISCUSSÃO

A integridade do sistema auditivo, periférico e central, é fundamental para desenvolver adequadamente a comunicação oral e escrita, uma vez que a linguagem compartilha mecanismos cognitivos subjacentes com as habilidades auditivas^(10)^. Autores afirmam que os primeiros anos de vida são considerados críticos para o desenvolvimento e, as experiencias auditivas vivenciadas neste período, estão diretamente ligadas ao desenvolvimento das habilidades da audição, tais como detecção, discriminação, localização, reconhecimento e compreensão auditiva^(11)^. Dessa forma, a maturação neuromuscular e do sistema sensorial de forma íntegra tem ligação direta com as habilidades de fala e linguagem. Quando se adquire um padrão articulatório de língua, há a habilidade auditiva de percepção de fala, pois os aspectos sensoriais e motores estão sendo abrangidos.

O mecanismo responsável pelas habilidades auditivas é o PAC, o qual desenvolve-se até, em média, de dez a 12 anos de idade^(11)^. Há fatores, como a integridade do sistema auditivo periférico e a maturação do sistema nervoso central, principalmente no que se refere às áreas auditivas primárias e secundárias que podem influenciar diretamente no desenvolvimento auditivo^(12)^. Esses fatores devem ser amplamente investigados para entendimento de possíveis alterações. Dessa forma, o roteiro buscou observar, de forma minuciosa, os principais fatores de risco para a alteração no PAC.

Durante a primeira infância, alterações periféricas e históricos de otite média secretora, podem comprometer a maturação das vias auditivas. Este comprometimento apresenta repercussão nas habilidades auditivas centrais e no processo de aprendizagem, por isso a necessidade de investigar o histórico auditivo do paciente^(10)^.

As questões sobre o desenvolvimento auditivo da criança, o histórico, se houve algum trauma, qual o histórico familiar e se fez/faz uso de antibióticos que possam ser ototóxicos estão abordados na parte quatro do roteiro nomeada por ‘’desenvolvimento auditivo”. Segundo a literatura, a etiologia dos transtornos de PAC inclui otites, febres altas e contínuas, distúrbios específicos do desenvolvimento da função auditiva, lesões nas vias de condução e privação sensorial durante a primeira infância^(13)^.

Todos esses fatores citados anteriormente irão influenciar, de forma efetiva, no desenvolvimento auditivo, na linguagem oral e escrita do paciente. As dificuldades encontradas podem ser observadas pelos familiares e cuidadores por meio de comportamentos, tais como dificuldades na realização de tarefas complexas ou mais longas, distração, sensibilidade a sons altos, dificuldade em seguir ordens verbais, repetição de estímulos verbais, dificuldade em compreender piada e linguagem figurada^(5)^. As queixas foram abordadas no roteiro, na parte três, pois a dificuldade em compreender a fala em presença de ruído de fundo, maior distratibilidade, atenção reduzida, dificuldade de comunicação e baixo desempenho acadêmico são sinais de risco para alterações do PAC^(13)^.

Os transtornos do processamento auditivo consistem em uma inabilidade de atentar, discriminar, reconhecer, recordar ou compreender informações auditivas^(11)^.

Com a apresentação de prejuízos nas áreas acima descritas, há a necessidade de uma avaliação aprofundada. É importante avaliar habilidades que fazem parte do desenvolvimento normal da linguagem, pois diversos distúrbios de linguagem oral podem ser detectados e acompanhados de forma precoce.

Na parte cinco, caracterizada como ‘’desenvolvimento de linguagem”, foram citados os principais marcos do desenvolvimento. Prejuízos na interpretação de informações sensoriais podem ser indicativos de alteração auditiva o que, futuramente, pode acarretar em um transtorno do processamento auditivo, e na aquisição da fala e linguagem^(12)^.

De acordo com a literatura pesquisada, não há um instrumento que observe a relação de linguagem e audição, em especial do PAC em pré-escolares, apenas em crianças em idade escolar. Foram pesquisados artigos e materiais sobre o tema nas bases de dados Lilacs, Sielo, PUBmed e acervo de livros e teses em biblioteca de instituição, no entanto foram observados apenas protocolos e roteiros que contemplavam crianças acima de seis anos de idade. Dessa forma, reafirma-se a importância da construção do material que visa o rastreio precoce de riscos e possíveis alterações de audição e linguagem.

O presente roteiro constituiu, portanto, um instrumento para ser utilizado na prática clínica para avaliações, triagens e acompanhamento de pré-escolares para indicar possíveis riscos para o transtorno do processamento da informação auditiva. O intuito do estudo é a detecção precoce dos possíveis transtornos para acompanhamento e estimulação das habilidades auditivas para que, no futuro, os prejuízos sejam sanados ou menores para a criança.

O roteiro faz parte de um estudo em andamento, que avaliará crianças menores de seis anos de idade. A pesquisa, até o presente momento, ainda não ocorreu por conta da pandemia do COVID-19.

## CONCLUSÃO

Foi elaborado um roteiro de pesquisa para realizar avaliação e indicar possíveis riscos para o transtorno do PAC em pré-escolares. O roteiro, Anexo A, é de extrema importância visto que não há na literatura instrumentos de triagem de PAC em pré-escolares que investiguem, de forma minuciosa, todo o processo que permeia o desenvolvimento auditivo e de linguagem de crianças de 43 a 47 meses.

Este período a ser investigado é crítico para o desenvolvimento, fatores citados, como otites, uso de medicamentos ototóxicos, entre outros, podem causar alteração no desenvolvimento auditivo, causam impacto no desenvolvimento da linguagem oral e escrita, e consequentemente na aprendizagem escolar.

## Anexo A. Roteiro de avaliação

1. IDENTIFICAÇÃO E ANAMNESE

Nome:

Idade:

Sexo feminino () Sexo masculino ()

Data de nascimento:

Naturalidade:

Telefones:

Escola: Período:

Preferência manual:

Com quem passa a maior parte do tempo:

Mãe: Idade:

Profissão:

Grau de escolaridade:

Tempo que fica com a criança por dia:

Pai: Idade:

Profissão:

Grau de escolaridade:

Tempo que fica com a criança por dia:

2. DADOS MATERNOS E DE GESTAÇÃO

Fuma () sim () não

Ingere bebida alcoólica () sim () não

Uso de medicamentos:

HIV () sim () não

Sífilis () sim () não

Hipertensão () sim () não

Diabetes () sim () não

Rubéola () sim () não

Herpes () sim () não

Toxoplasmose () sim () não

Doenças cardiovasculares () sim () não

Doenças renais () sim () não

Internação durante a gestação:

Intercorrências:

Quantas semanas de gestação a criança nasceu:

Observações:

3. QUEIXAS

Apresenta queixas relacionadas a agitação?

Apresenta queixas relacionadas a desatenção?

Apresenta dificuldade para ouvir e entender em ambientes ruidosos?

Apresenta dificuldade para se relacionar com as pessoas?

Apresenta dificuldades para realizar atividades motoras globais?

Apresenta dificuldades para realizar atividades motoras finas?

4. DESENVOLVIMENTO AUDITIVO

Histórico de otites recorrentes na primeira infância?

Antecedentes otológicos?

Sente dores de ouvido frequentemente **(otalgia)?**

Já teve algum trauma na orelha?

Uso de antibióticos? **Quando e por quanto tempo?**

Histórico familiar?

5. DESENVOLVIMENTO DE LINGUAGEM

Faz combinação de três ou mais palavras?

Identifica partes do corpo?

Compreende instruções que lhe são direcionadas?

Reconhece pronomes que diferenciam os sexos?

Reconhece adjetivos, como ‘’grande”, ‘’pequeno”, ‘’feliz”?

Faz uso de artigos, como ‘’o”, ‘’a”, ‘’um”, ‘’uma”?

Faz uso de plurais, como ‘’bala-balas”?

Faz uso de preposições, como ‘’com”, ‘’de”, ‘’para”?

Faz uso de verbos auxiliares, como ‘’ ter”, ‘’ser”, ‘’estar”?

Compreende conceitos abstratos?

Compreende conceito de quantidade?

Faz deduções?

Formula perguntas?

Solicita ações e objetos, como dar um brinquedo ou pegar água?

Solicita permissão, com ‘’posso”?

Usa a negação?

Relata acontecimentos do passado ou antecipa do futuro?

Compreende e consegue recontar histórias com turnos narrativos?

Mantem tópico de conversação aguardando seu turno?

Canta músicas?

6. DESENVOLVIMENTO MOTOR

O desenvolvimento motor apresenta-se adequado para a idade?

7. AVALIAÇÃO SIMPLIFICADA DO PROCESSAMENTO AUDITIVO CENTRAL (ASPA)

Nome:

Idade:

Avaliador:

Data de avaliação:

1. Teste de localização sonora

Desempenho

() direita () esquerda () em cima () à frente () atrás

Critério de normalidade

≥ 4 acertos incluindo a direita e a esquerda

Resultado: _____

**Table:**

	Acertos	Normal	Alterado
Teste de localização sonora			

Avaliação da habilidade auditiva de localização sonora:

() normal () alterada

2. Teste de memória para sons verbais ou não verbais em sequência

2.1 TMSV em sequência (com três sons):

Produção fonoarticulatória isolada da sílaba () PA () TA () CA

Desempenho:

**Table:**

	Sim	Não
PA TA CA		
TA PA CA		
CA TA PA		

Critério de normalidade

≥ 2 acertos para TMSV em 3 tentativas (≥2/3)

Resultado: ____ (com três sons)

Conclusão

**Table:**

	Acertos	Normal	Alterado

Avaliação da habilidade auditiva de ordenação temporal:

() normal () alterada

Outros comportamentos observados durante a realização deste teste:

() capacidade de atenção inadequada

() capacidade de memória inadequada

() atitude motora inadequada

() dificuldade de compreender as solicitações

() cansa-se facilmente

Observações:___________________________________________________________________________________________________________________________

Para o teste de localização sonora, é colocado como critério de normalidade, acima de quatro acertos. O avaliador deverá marcar, em protocolo específico, o número de acertos e se está normal ou alterado (de acordo com a pontuação alcançada). Para o teste de sequência para sons verbais deverá haver ao menos dois acertos nas três sequências nomeadas como critério de normalidade, assim como para sons não verbais, em que deverá haver também dois acertos em três tentativas. Com os achados dos três testes, o avaliador concluirá se há alteração de Processamento Auditivo central.

Adequado: () sim () não

8. AVALIAÇÃO AUDIOLÓGICA COMPORTAMENTAL:

Observado através do comportamento auditivo da criança diante do estimulo instrumental.

**Table:**

Instrumento	Intensidade	Resposta
Tambor	Fraca ()	
	Média ()	
	Forte ()	
Chocalho	Fraca ()	
	Média ()	
	Forte ()	
Guizo	Fraca ()	
	Média ()	
	Forte ()	
Reco-Reco	Fraca ()	
	Média ()	
	Forte ()	
Agogô (duas cânulas)	Fraca ()	
	Média ()	
	Forte ()	

Adequado: () sim () não

Reflexo cócleo-palpebral para sons intensos (tambor e agogô)

Adequado: () sim () não
